# Influence of Nature Support on Methane and CO_2_ Conversion in a Dry Reforming Reaction over Nickel-Supported Catalysts

**DOI:** 10.3390/ma12111777

**Published:** 2019-05-31

**Authors:** Anis Hamza Fakeeha, Samsudeen Olajide Kasim, Ahmed Aidid Ibrahim, Ahmed Elhag Abasaeed, Ahmed Sadeq Al-Fatesh

**Affiliations:** Chemical Engineering Department, College of Engineering, King Saud University, P.O. Box 800, Riyadh 11421, Saudi Arabia; sofkolajide2@gmail.com (S.O.K.); aidid@ksu.edu.sa (A.A.I.); abasaeed@ksu.edu.sa (A.E.A.); aalfatesh@ksu.edu.sa (A.S.A.-F.)

**Keywords:** methane reforming, greenhouse gas, Ni-supported catalysts, carbon deposition, catalytic activity

## Abstract

A promising method to reduce global warming has been methane reforming with CO_2_, as it combines two greenhouse gases to obtain useful products. In this study, Ni-supported catalysts were synthesized using the wet impregnation method to obtain 5%Ni/Al_2_O_3_(SA-5239), 5%Ni/Al_2_O_3_(SA-6175), 5%Ni/SiO_2_, 5%Ni/MCM41, and 5%Ni/SBA15. The catalysts were tested in dry reforming of methane at 700 °C, 1 atm, and a space velocity of 39,000 mL/gcat h, to study the interaction of Ni with the supports, and evaluation was based on CH_4_ and CO_2_ conversions. 5%Ni/Al_2_O_3_(SA-6175) and 5%Ni/SiO_2_ gave the highest conversion of CH_4_ (78 and 75%, respectively) and CO_2_ (84 and 82%, respectively). The catalysts were characterized by some techniques. Ni phases were identified by X-ray diffraction patterns. Brunauer–Emmett–Teller analysis showed different surface areas of the catalysts with the least being 4 m^2^/g and the highest 668 m^2^/g belonging to 5%Ni/Al_2_O_3_(SA-5239) and 5%Ni/SBA15, respectively. The reduction profiles revealed weak NiO-supports interaction for 5%Ni/Al_2_O_3_(SA-5239), 5%Ni/MCM41, and 5%Ni/SBA15; while strong interaction was observed in 5%Ni/Al_2_O_3_(SA-6175) and 5%Ni/SiO_2_. The 5%Ni/Al_2_O_3_(SA-6175) and 5%Ni/SiO_2_ were close with respect to performance; however, the former had a higher amount of carbon deposit, which is mostly graphitic, according to the conducted thermal analysis. Carbon deposits on 5%Ni/SiO_2_ were mainly atomic in nature.

## 1. Introduction

The abundant natural gas in large reserves positions methane as a good candidate, ahead of any other, for the chemistry of one-carbon-atom-containing compounds [1]. Consequently, the burning of natural gas, as well as other fossil fuels, to cater for the demand for energy results in a significant release of CO_2_ to the atmosphere and leads to global warming. Therefore, good use of the two agents CO_2_ and natural gas, (i.e., dry reforming of methane), will ultimately reduce the earth warming effect being caused by them.

With the great development recorded in modern science and technology, the change in energy structure and the creation of a benign environment have become imperative to achieve development that is sustainable. The dry reforming of methane (DRM) will not only reduce the environmental problem that arises, but also lead to the production of valuable feedstock that can be used to synthesize other important chemicals through Fischer–Tropsch synthesis [2,3,4,5]. DRM gives syngas mixtures with a low ratio of H_2_/CO in comparison with steam reforming of methane [6,7]. In recent times, DRM has attracted a lot of attention due to its ability to convert the two global warming agents into useful products; however, the lack of stable and selective catalysts for the process, coupled with the high endothermic nature of the reaction, throw a spanner in the works for the commercialization of the process [8].

There are challenges associated with the study of the DRM reaction. Some of these are thermal sintering, oxidation of metal, and coking [9]. The latter would encapsulate catalysts’ active sites and gradually makes the catalysts inactive. Thermal sintering and metal oxidation would lead to direct deactivation of catalysts. It has been observed that active metals and supports play a crucial role in catalyst performance during DRM [10]. Noble metals, such as Pt, Ru, Rh, and many others, have a superior tendency to suppress coking, i.e., carbon deposition [11,12], but the expensive nature of these metals puts a restriction on their application at the industrial level.

On the one hand, nickel catalysts that have an appreciably high performance and low cost have been extensively used in DRM. However, at high temperatures, they are less resistant to sintering and suffer from coking, which may gradually lead to the deactivation of the catalysts in the course of the reaction. According to the literature, there are several methods that can be employed to suppress carbon deposition and sintering of nickel nanoparticles, possibly by using supports with basic properties [13,14,15], ensuring better dispersion of active metal nanoparticles on the support [16], doping with noble metals as promoters [17,18], and enhancing the interaction between metal and support [19,20].

In this study, Ni-catalysts supported on a metal oxide, such as Al_2_O_3_(SA-5239), Al_2_O_3_(SA-6175), SiO_2_, MCM41, and SBA15, were synthesized and employed in the DRM. The effects of the different metal oxide supports were investigated in terms of their interaction with Ni metal, the catalysts’ stability, and activity in methane reforming with CO_2_.

## 2. Results and Discussion

### 2.1. Characterization of Fresh Catalysts

#### 2.1.1. Surface Characterization

**Figure 1:**
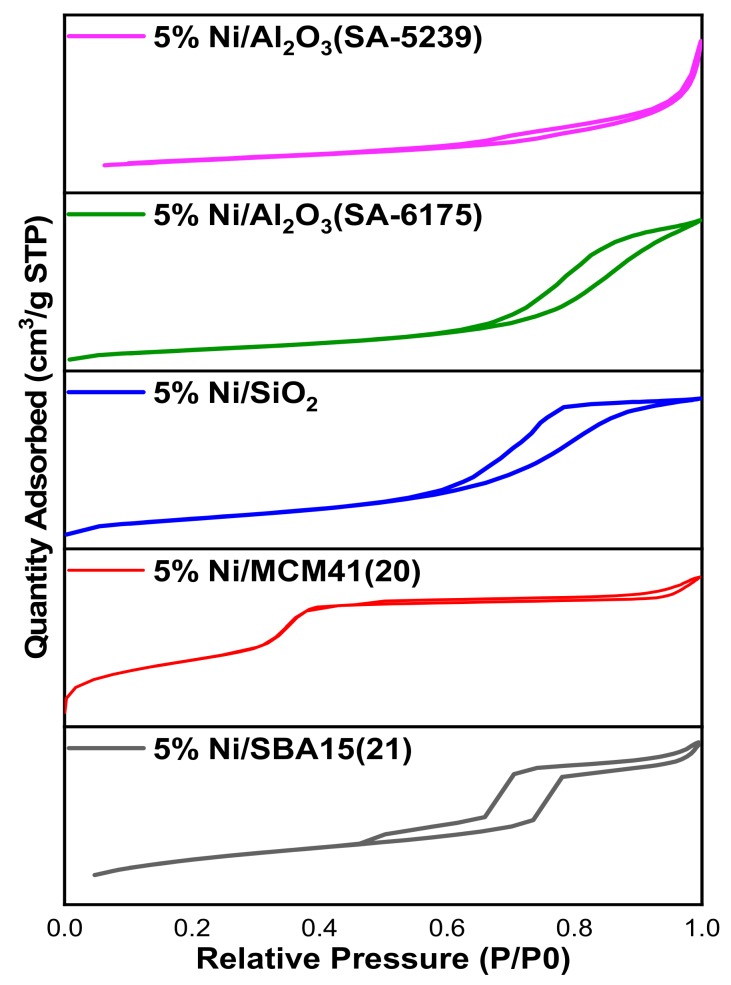
N_2_ Adsorption-desorption isotherms for the Ni-supported catalysts.

For the textural properties, the results obtained from the N_2_ physisorption are shown in Table 1, and that of the isotherms are presented in Figure 1. The results explain the differences in the activities of the catalysts. According to the results of the N_2_ physisorption presented in the Table 1, 5%Ni/Al_2_O_3_(SA-5239) has the lowest specific surface area, while 5%Ni/SBA15 has the highest of about 668 m^2^/g.

Figure 1 shows the N_2_ adsorption-desorption isotherms. In accordance with the IUPAC (International Union of Pure and Applied Chemistry) classification of isotherms, the isotherms for the fresh catalyst samples fall under the category of type IV and V, which are indicative of mesoporous materials [21]. It can be inferred from the figure(s) that 5%Ni/SBA15 and 5%Ni/MCM41 have isotherms belonging to the type IV category, while the remaining samples have isotherms similar to the type V category. The sharp increase in adsorption for Ni-supported on 5%Ni/SBA15, observed at relative pressure of about 0.73, established the fact that the well-ordered hexagonal mesoporous frameworks of SBA15 were maintained after the impregnation step [22].

The most probable diameters for the samples from the BJH (Barrett, Joyner, and Halenda) pore size distribution curves are listed in Table 1. All the values fall within the range of mesoporous materials.

#### 2.1.2. X-ray Diffraction (XRD)

**Figure 2:**
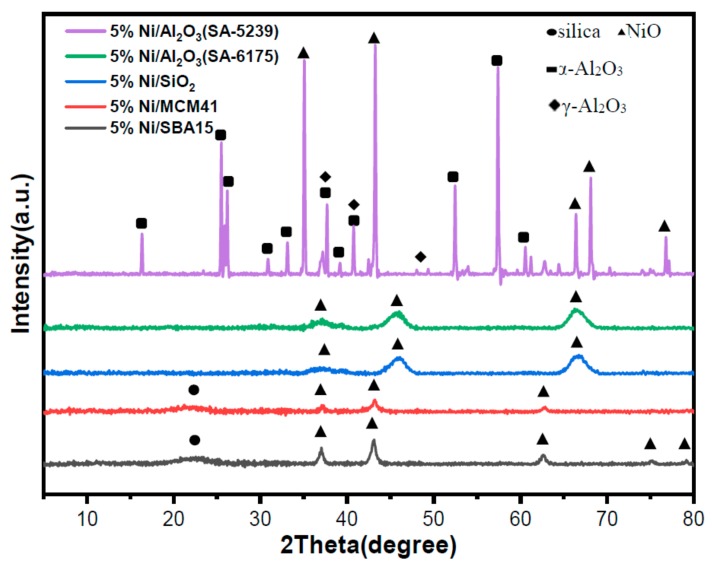
XRD patterns for the freshly calcined catalysts. (a.u.: arbitrary unit).

The phase composition of the freshly calcined catalysts was studied by performing XRD analysis. The XRD crystallography obtained is shown in Figure 2. From Figure 2, it can be inferred that NiO particles were well-dispersed in all of the samples, except for 5%Ni/Al_2_O_3_(SA-5239). A characteristic peak, attributed to amorphous phase of silica, was observed at around 23° [23] in 5%Ni/MCM41 and 5%Ni/SBA15, because these two samples contain certain proportions of silica. NiO phases can be identified on the catalysts at 2*θ* angles around 36°, 43°, 46°, 63°, 66°, 75°, and 78°. Similar findings were reported by Yu et al., in their study of electrocatalytic activity for methanol oxidation using Ni-NiO@C nanocomposites synthesized by a simple solution-combustion method [24]. The additional peaks that appeared on 5%Ni/Al_2_O_3_(SA-5239) could be assigned to α-gamma alumina, and the low surface area support that was used in the synthesis of the catalyst. Kelekanjeri et al. reported similar observations in their research on using the combustion chemical vapor deposition method for depositing α-alumina [25].

#### 2.1.3. H_2_-Temperature Programmed Reduction (H_2_-TPR)

**Figure 3:**
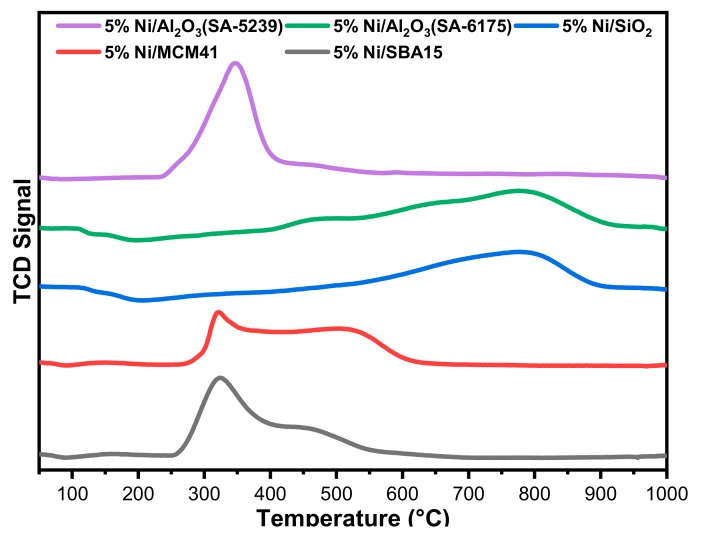
Temperature-Programmed Reduction (TPR) profiles for the Ni-supported catalysts.

Figure 3 shows the reduction behavior of the freshly calcined catalysts. H_2_-TPR profiles reveal the reducibility and the extent of interaction of the active metal (Ni) with the different supports. The reduction profiles of the catalysts 5%Ni/Al_2_O_3_ (SA-5239), 5%Ni/SBA15, and 5%Ni/MCM41 are characterized by a sharp reduction peak at temperature 350, 328, and 325 °C, respectively. Each one of them has a shoulder within the temperature range 450–550 °C. 5%Ni/Al_2_O_3_(SA-6175) has a broad peak with three less pronounced peaks at 475, 650, and 780 °C. 5%Ni/SiO_2_ has a single wide peak at 770 °C.

In general, the reduction peak for Ni-supported catalysts in the temperature range of 400–500 °C could be attributed to NiO species that have weak interaction with the support. The peaks that appear at 500–600 °C could be assigned to NiO species having a medium strength of interaction. Lastly, peaks appearing at temperatures above 600 °C are assigned to the species that have strong interaction with the support [26]. Strong interaction of NiO with the supports was observed for 5%Ni/Al_2_O_3_(SA-6175) and 5%Ni/SiO_2_. Such catalyst samples as 5%Ni/Al_2_O_3_ (SA-5239), 5%Ni/SBA15, and 5%Ni/MCM41 will relatively be easy to reduce compared to 5%Ni/Al_2_O_3_(SA-6175) and 5%Ni/SiO_2,_ in which the NiO species interacted strongly with the support.

#### 2.1.4. CO_2_-Temperature Programmed Desorption (CO_2_-TPD)

**Figure 4:**
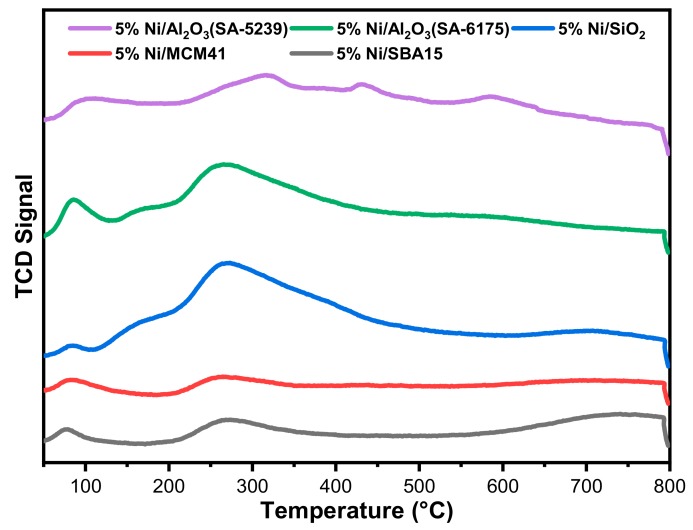
CO_2_-Temperature Programmed Desorption (CO_2_-TPD) profiles for the freshly calcined catalysts.

CO_2_-TPD analysis was carried out to investigate the basic nature of the different catalyst samples. The results obtained from the analysis can be seen in Figure 4.

Catalysts’ basicity has a significant effect on their activity in DRM, owing to the acidic nature of carbon dioxide. Hence, strong basic sites could improve catalytic activity by enhancing the chemisorption of reacting gases [27]. The basic sites on a catalyst are usually classified as weak, intermediate, strong, and very strong at the desorption temperature ranges of 20–150, 150–300, 300–450, and >450 °C, respectively [28,29].

From the TPD profiles, the catalysts are characterized by four peaks (the fourth is not so pronounced), except for 5%Ni/SBA15 and 5%Ni/MCM41, which have only two peaks. Virtually all the catalyst samples have a similar basic site classification. For the samples with four peaks, three of their peaks fall under the category of weak and intermediate basic sites, while the fourth, in each case, falls in the very strong basic site classification. 5%Ni/Al_2_O_3_(SA-5239) has its third peak in the strong region.

Both 5%Ni/SBA15 and 5%Ni/MCM41 have their peaks in the weak and intermediate regions.

### 2.2. Catalyst Performance

**Figure 5:**
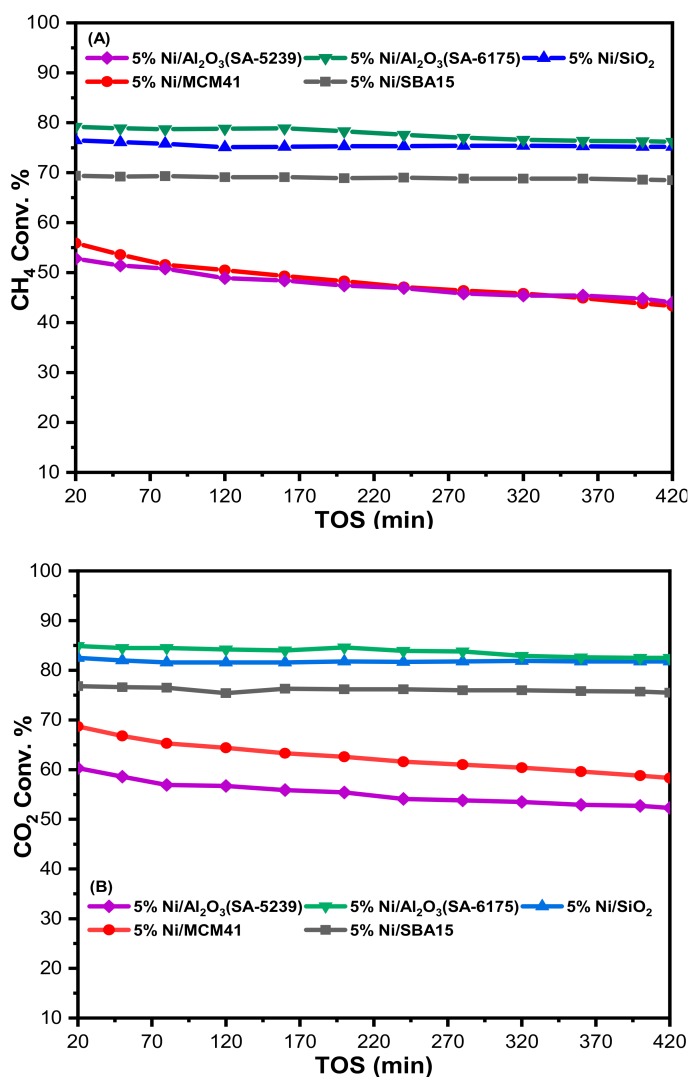

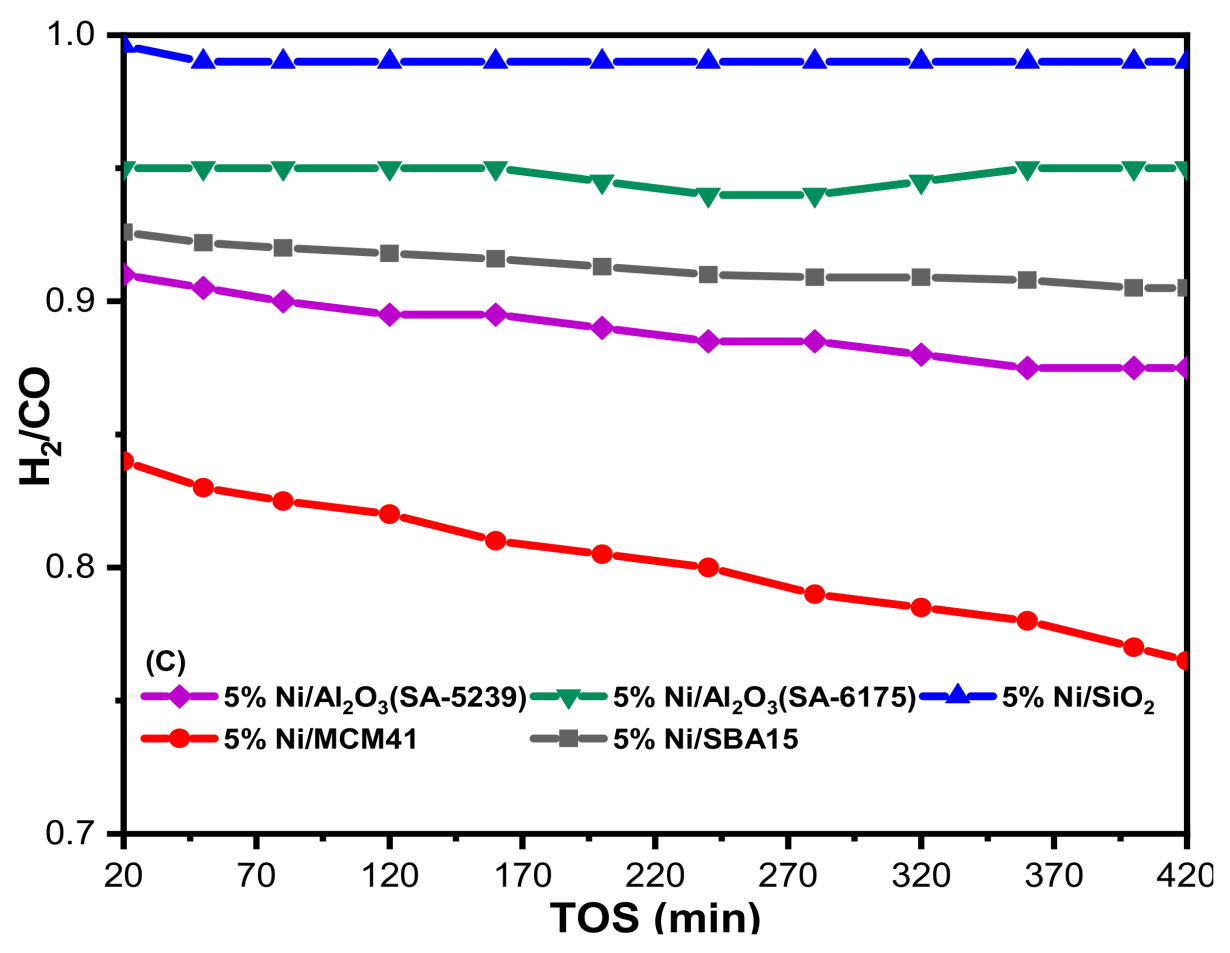
(**A**) CH_4_ conversion, (**B**) CO_2_ conversion, and (**C**) H_2_/CO ratio at atmospheric pressure, 700 °C, and GHSV = 39,000 mL/gcat h. (TOS: time on stream, GHSV: gas hourly space velocity).

Catalyzed DRM has been carried out at 700 °C, at atmospheric pressure and a gas hourly space velocity (GHSV) of 39,000 mL/gcat h over Ni-catalysts with different supports. As shown in Figure 5, the supports play a pivotal role in the performance of the Ni-based catalysts during the dry reforming reaction. This could be due to the difference in the metal dispersion and the strength of interaction of Ni with the different supports, all of which are dependent on the nature of the supports [30]. 

The effects of these supports have been reported in terms of CH_4_ and CO_2_ conversion using time on stream (TOS). From Figure 5, none of the catalysts showed fast deactivation, which suggests that there was no sintering of the active metal. On the other hand, 5%Ni/Al_2_O_3_(SA-5239) and 5%Ni/MCM41 had the lowest CH_4_ conversion of about 48%. The poor performance of the former could be attributed to its low BET (Brunauer–Emmett–Teller) surface area, which implies that the active metal was poorly dispersed on the support. On the contrary, 5%Ni/MCM41 has a high surface area and yet had a low CH_4_ conversion. This behavior may be because of the catalyst’s weak basic sites, which have low intensity. This will inhibit the CO_2_ chemisorption ability of the catalyst and variably affects reforming of methane. Moreover, the active metal in both 5%Ni/Al_2_O_3_(SA-5239) and 5%Ni/MCM41 has weak interaction with the supports according to the low reduction temperature revealed by the H_2_-TPR results. The highest CH_4_ conversion was observed for both 5%Ni/SiO_2_ and 5%Ni/Al_2_O_3_(SA-6175) at about 75 and 78%, respectively. The two catalysts have an appreciably high surface area and the active metal has strong interaction with their support, as revealed by the TPR profiles.

The same trend was observed for CO_2_ conversion, as shown in Figure 5B. 5%Ni/Al_2_O_3_(SA-5239) and 5%Ni/MCM41 have the lowest conversion of about 55 and 63%, respectively. 5%Ni/SBA15 maintained an intermediate position, as observed in Figure 5A, and had CO_2_ conversion of about 76%. Ni supported on Al_2_O_3_(SA-6175) had the highest CO_2_ conversion, averaging 84% over the considered time on stream. For all of the catalysts investigated, it was evident that CO_2_ conversion was always higher than the CH_4_ conversion. This is suggestive of the occurrence of a reverse water gas shift (RWGS) reaction

(1) $$H_{2}O+CO⇌H_{2}+CO_{2}.$$

The results (Figure 5C) obtained from the H_2_ and CO yield for each of the catalysts further strengthen the above claim. From the figure, all the catalysts had a H_2_/CO ratio of less than 1.

### 2.3. Spent Catalyst Characterization

#### 2.3.1. Temperature-Programmed Oxidation (TPO)

One of the useful techniques that can be used to determine the nature of the carbon that is deposited onto catalysts’ surface is TPO. According to the literature, carbon deposited onto the surface of catalysts in dry reforming reactions exists in different forms; we have atomic carbon, graphitic carbon, and amorphous carbon. In an oxidative atmosphere, and at a different temperature, the carbon can undergo gasification to form CO_2_. Carbon that undergoes gasification at temperatures below 250 °C is termed atomic carbon, while the carbon gasified within 250–600 °C is classified as amorphous. Meanwhile graphitic carbon is gasified at temperatures above 600 °C [31].

**Figure 6:**
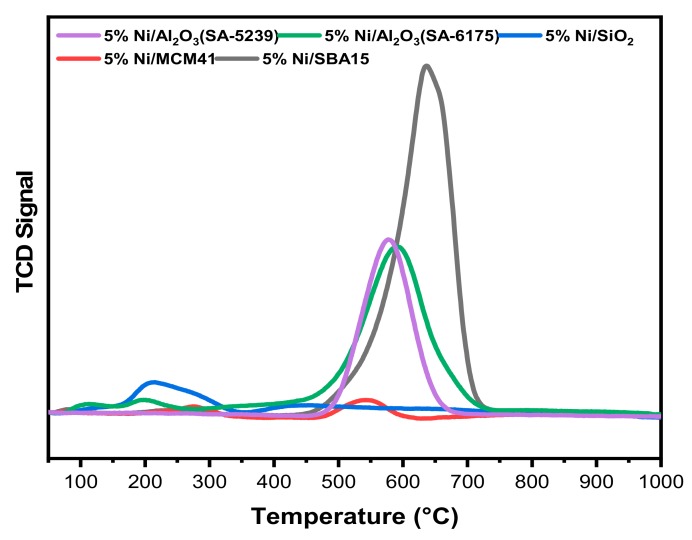
Temperature-Programmed Oxidation (TPO) profiles for all of the spent catalysts.

The TPO profiles for the spent catalysts are shown in Figure 6. From Figure 6, 5%Ni/Al_2_O_3_ (SA-5239), 5%Ni/Al_2_O_3_ (SA-6175), and 5%Ni/SBA15 exhibited similar behavior, by showing a single sharp peak. Both 5%Ni/Al_2_O_3_ (SA-5239) and 5%Ni/Al_2_O_3_ (SA-6175) contain amorphous carbon, with the latter having a small amount of atomic carbon according to the shoulder observed at a temperature below 200 °C. According to the profile of 5%Ni/SBA15, the only peak it has appeared around 640 °C, which suggests that the carbon deposit has a graphitic nature. 5%Ni/SiO_2_ showed the presence of a mixture of the three forms of carbon deposit, as its peaks are seen to overlap over the temperature range. However, the broad peak at a low temperature range shows that the carbon deposits mostly comprise atomic carbon. 5%Ni/MCM41 exhibited two sharp peaks, one at 280 °C and the other at 550 °C, which are within the range of amorphous carbon.

#### 2.3.2. Thermo-Gravimetric Analysis (TGA)

**Figure 7:**
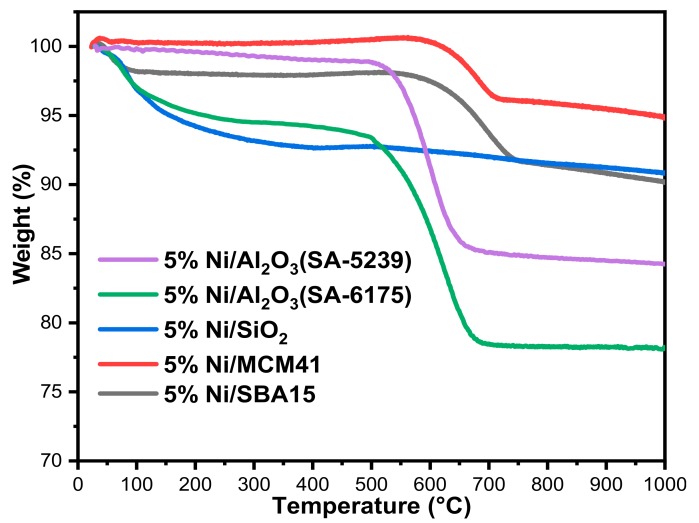
Curves showing the quantitative analysis of the deposited carbon on the used catalysts.

At the end of the 7 h reaction, the spent catalysts were analyzed by TGA, which is a quantitative technique that reveals the quantity of carbon deposits. Figure 7 shows the results of the analysis. The percentage loss of weight for all of the spent samples began at different temperatures. As seen in Figure 7, 5%Ni/MCM41 had the lowest carbon deposit of about 5%. Next in line were both 5%Ni/SBA15 and 5%Ni/SiO_2,_ which had weight loss of ~9%. 5%Ni/SiO_2_ maintained a constant weight at around 340 °C up until the end of the analysis, despite its high activity. As revealed by the TPO, the carbon deposit on 5%Ni/SiO_2_ is mostly atomic, which can easily be gasified. Meanwhile, its closest counterpart in activity had the highest weight loss of about 22%. It maintained a constant weight at approximately 680 °C, which is just sufficient for the gasification of the form of carbon deposited on it according to Figure 6.

### 2.4. Scanning Electron Microscope (SEM)

**Figure 8:**
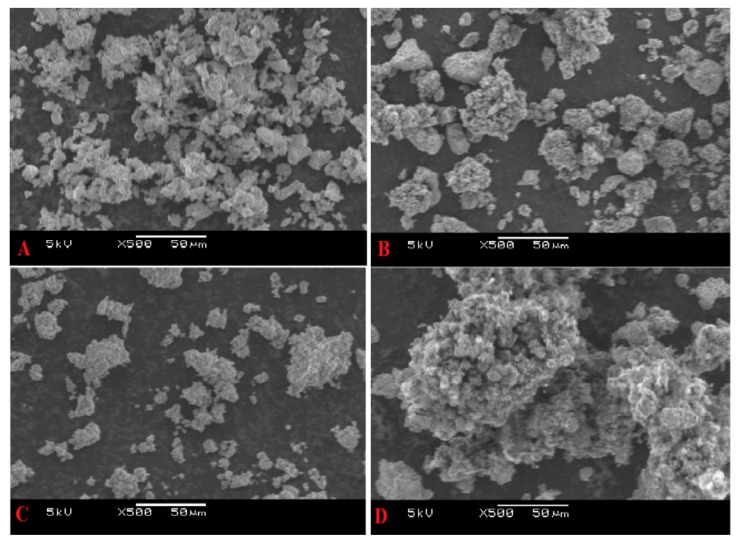
Scanning Electron Microscope (SEM) images for (**A**) fresh 5%Ni/Al_2_O_3_ (SA-5239), (**B**) used 5%Ni/Al_2_O_3_ (SA-5239), (**C**) fresh 5%Ni/Al_2_O_3_ (SA-6175), and (**D**) used 5%Ni/Al_2_O_3_ (SA-6175).

The SEM was used to see what changes have taken place on the surface morphology of both 5%Ni/Al_2_O_3_ (SA-5239) and 5%Ni/Al_2_O_3_ (SA-6175) by comparing the surface images of the fresh and used samples. The images obtained from the machine are as shown in Figure 8. The particles virtually have the same morphology; the difference lies in the fact that more agglomeration was observed for the sample having a higher BET surface area, i.e., 5%Ni/Al_2_O_3_ (SA-6175).

## 3. Materials and Methods

### 3.1. Catalyst Preparation

All of the Nickel (Ni)-supported catalysts that were investigated in this research work were prepared using the wet impregnation technique. Some of the supports were purchased from chemical manufacturing industries, while the others were synthesized. Al_2_O_3_ (SA-5239) (CAS 1344-28-1) and Al_2_O_3_ (SA-6175) (CAS 7429-90-5) were purchased from Norton Chemical Industries (Akron, OH, USA), and SiO_2_ from Fisher Scientific Company (Akron, OH, USA). MCM41 and SBA15 were prepared by the procedure explained in [32,33]. Hydrated Ni nitrate [Ni(NO_3_)_2_·6H_2_O] (CAS 13621) was used as the active metal for all the supports, and the percentage loading was maintained at 5 wt.% in all samples.

Calculated amounts of the active metal were weighed and dissolved in 30 mL of distilled water and subsequently followed by the addition of the supports in separate crucibles. Each of the mixtures (i.e., support-active metal), in a separate crucible, was placed over a hot plate with the temperature set at 80 °C for 3 h with continuous stirring. Thereafter, the slurry was placed in a furnace for drying at 120 °C overnight. The dried samples were calcined at 550 °C for 3 h in the same furnace.

### 3.2. Characterization

The Brunauer–Emmet–Teller technique was used in calculating the specific surface area of the samples with the aid of a device that analyzes the surface area and porosity, i.e., a Micromeritics Tristar II 3020 (Micromeritics, Norcross, GA, USA). To perform nitrogen physisorption measurements, 0.20–0.30 g of the samples were weighed and subjected to degassing at 200 °C for three hours prior to the analysis.

X-ray powder diffraction patterns for the catalyst samples were recorded with a Rigaku (Miniflex) diffractometer, (Rigaku Corporation, The Woodlands, TX, USA) employing a Cu K_α_ radiation source and a nickel filter, operated at 40 kV and 40 mA. The step size and scanning range of 2*θ* for analysis were set to 0.01° and 5–80°, respectively. Standard powder XRD cards (JCPDS) were used to document the available phases.

The reduction behavior of the fresh catalysts was determined with the AutoChem II (Micromeritics). A sample weight of 75.0 mg was analyzed. Heating of samples was carried out under pure Ar at 150 °C for half an hour, thereafter cooled to 25 °C. Afterwards, samples were heated to 1000 °C at 10 °C/min by allowing the flow of 10% H_2_/Ar gas at 40 mL/min. A thermal conductivity detector (TCD) was used to observe the H_2_ consumption. Temperature-programmed desorption of carbon dioxide (CO_2_-TPD) and CO pulse chemisorption measurements were obtained using automatic chemisorption equipment (Micromeritics AutoChem II 2920) with a TCD. At the start, a 70 mg sample was heated at 200 °C for 1 h under a helium (He) flow to remove adsorbed components. Then, CO_2_ adsorption was carried out at 50 °C for 60 min in the flow of a He/CO_2_ gas mixture (90:10 volume ratio) at 30.0 mL/min. Afterwards, an increase in temperature up to 800 °C at 10 °C/min was registered as the CO_2_ desorption signal by the TCD.

Carbon deposition over the used catalysts was measured by doing thermal analysis under atmospheric condition with the aid of TGA-51 (Shimadzu, Kyoto, Japan) equipment. A certain amount from the spent catalyst (10 mg) was subjected to heat treatment within the temperature range 25–1000 °C. The ramping temperature was maintained at 20 °C/min.

TPO was performed in an oxidative atmosphere to determine the kind of carbon deposited over the surface of the catalyst using the Micromeritics AutoChem II over a temperature range of 50–800 °C, under the flow of a 10% O_2_/He mixture at 40 mL/min. The spent catalyst was first pretreated in the presence of high-purity Argon at 150 °C for 30 min, and subsequently cooled to room temperature.

Catalysts’ morphology was studied using the JEOL JSM-7100F (JEOL Ltd., Tokyo, Japan) field emission SEM.

### 3.3. Catalyst Activity

The DRM was carried out in a stainless steel fixed-bed tubular micro-reactor (ID = 9 mm) at atmospheric pressure. The reactor system was procured from Process Integral Development (PID Eng. & Tech). Before the start of the DRM reaction, 0.10 g of catalyst was activated by a H_2_ flow of 40 mL/min at 700 °C for 60 min. N_2_ gas was then let into the reactor for 20 min to remove the remnants of H_2_ while the system was maintained at the reaction temperature (700 °C). Thereafter, feed gases of CH_4_, CO_2_, and N_2_ were injected at the molar ratio of 6:6:1, respectively, at a 65 mL/min total flow rate. The temperature, pressure, and reaction variables were inspected through the reactor panel. A GC GC-2014 Shimadzu, unit having a TCD and two columns, Porapak Q and Molecular Sieve 5A, were connected in series/bypass connections in order to completely analyze the reaction products. The following equations were used to calculate the CH_4_ and CO_2_ conversions, respectively.

(2) $${\%\mathrm{CH}}_{4}\;\mathrm{conversion}=\;\frac{{\mathrm{CH}}_{4}\;\mathrm{in}-{\mathrm{CH}}_{4}\;\mathrm{out}}{{\mathrm{CH}}_{4}\;\mathrm{in}}\;\times \;100$$

(3) $${\%\mathrm{CO}}_{2}\;\mathrm{conversion}=\;\frac{{\mathrm{CO}}_{2}\;\mathrm{in}-{\mathrm{CO}}_{2}\;\mathrm{out}}{{\mathrm{CO}}_{2}\;\mathrm{in}}\;\times \;100$$

## 4. Conclusions

In this research, Ni-based catalysts were synthesized using the wet impregnation method and tested for DRM. Different supports were used in the synthesis, aimed at obtaining support that would give the best metal–support interaction. The synthesized catalysts were evaluated in terms of CH_4_ and CO_2_ conversion. From the results of the investigation, 5%Ni/Al_2_O_3_(SA-6175) and 5%Ni/SiO_2_ gave the highest CH_4_ conversion, averaging about 78 and 75%, respectively. The same trend was observed for the CO_2_ conversion, with 5%Ni/Al_2_O_3_(SA-6175) having 84%, while 5%Ni/SiO_2_ had 82% conversion. CO_2_ conversion was observed to be higher than CH_4_ conversion for all the catalysts.

Both fresh and spent catalysts were put through some characterizations. According to the BET method, the catalysts possess isotherms belonging to the type IV and V class, which are mesoporous in nature. The TPR profiles showed that Ni interacted weakly with the supports in 5%Ni/Al_2_O_3_ (SA-5239), 5%Ni/SBA15, and 5%Ni/MCM41, relative to 5%Ni/Al_2_O_3_(SA-6175) and 5%Ni/SiO_2_. The TPO profile showed similar behavior among 5%Ni/Al_2_O_3_(SA-5239), 5%Ni/Al_2_O_3_(SA-6175), and 5%Ni/SBA15, which had a sharp single peak that appeared at different temperatures. The former two were mostly characterized by amorphous carbon, while the latter has a graphitic carbon deposit. Two distinct peaks were observed for 5%Ni/SiO_2_ and 5%Ni/MCM41. The carbon deposit in 5%Ni/SiO_2_ was mostly atomic, while that of 5%Ni/MCM41 was comprised mainly of amorphous carbon. 5%Ni/MCM41 had the lowest carbon deposit, while 5%Ni/Al_2_O_3_(SA-6175) had the highest deposit, probably due to its high activity, according to the TGA.

## Figures and Tables

**Table 1:** N_2_ physisorption results for the different catalysts.

Catalyst	Surface Area (m^2^/g)	P.V (cm^3^/g)	P.D (nm)
5%Ni/Al_2_O_3_(SA-5239)	4	0.01	11.5
5%Ni/Al_2_O_3_(SA-6175)	209	0.68	11.6
5%Ni/SiO_2_	258	0.60	8.2
5%Ni/MCM41	583	0.64	3.7
5%Ni/SBA15	668	0.06	6.6

P.V = Pore Volume, P.D = Pore Diameter.

## References

[1] Caballero A., Perez P.J. (2013). Methane as raw material in synthetic chemistry: The final frontier. Chem. Soc. Rev..

[2] Olsbye U. (2016). Single-pass catalytic conversion of syngas into olefins via methanol. Angew. Chem. Int. Ed..

[3] Venvik H.J., Yang J. (2017). Catalysis in microstructured reactors: Short review on small-scale syngas production and further conversion into methanol, DME and Fischer-Tropsch products. Catal. Today.

[4] Ali K.A., Abdullah A.Z., Mohamed A.R. (2015). Recent development in catalytic technologies for methanol synthesis from renewable sources: A critical review. Renew. Sustain. Energy Rev..

[5] Hu J., Yu F., Lu Y. (2012). Application of Fischer-Tropsch synthesis in biomass to liquid conversion. Catalysts.

[6] Liu Q.G., Fangna L., Xiaopeng L., Youjun L., Huifang Z., Ziyi X., Guangwen S., Fabing S. (2014). Enhanced catalytic performances of Ni/Al_2_O_3_ catalyst via addition of V_2_O_3_ for CO methanation. Appl. Catal. A.

[7] Hu Y., Ruckenstein E. (2002). Binary MgO-based solid solution catalysts for methane conversion to syngas. Catal. Rev..

[8] Moghaddam S.V., Rezaei M., Meshkani F., Daroughegi R. (2018). Carbon dioxide methanation over Ni-M/Al_2_O_3_ (M: Fe, CO, Zr, La and Cu) Catalysts synthesized using the one-pot sol-gel synthesis method. Int. J. Hydrogen Energy.

[9] Xu L., Song H., Chou L. (2012). One-Pot synthesis of ordered mesoporous NiO-CaO-Al_2_O_3_ composite oxides for catalyzing CO2 reforming of CH_4_. ACS Catal..

[10] Khavarian M., Chai S.P., Mohamed A.R. (2015). The effects of process parameters on carbon dioxide rreforming of methane over Co-Mo-MgO/MWCNTs nanocomposite catalysts. Fuel.

[11] Jaiswar V.K., Katheria S., Deo G., Kunzru D. (2017). Effect of Pt doping on activity and stability of Ni/MgAl_2_O_4_ catalyst for steam reforming of methane at ambient and high pressure condition. Int. J. Hydrogen Energy.

[12] Bitter J.H., Seshan K., Lercher J.A. (1998). Mono and bifunctional pathways of CO_2_/CH_4_ reforming over Pt and Rh based catalysts. J. Catal..

[13] Li X., Li D., Tian H., Zeng L., Zhao Z.J., Gong J. (2017). Dry reforming of methane over Ni/La_2_O_3_ nanorod catalysts with stabilized Ni nanoparticles. Appl. Catal. B Environ..

[14] Abdullah B., Ghani N.A.A., Vo D.V.N. (2017). Recent advances in dry reforming of methane over Ni-based catalysts. J. Clean Prod..

[15] Li S., Tang H., Gong D., Ma Z., Liu Y. (2017). Loading Ni/La_2_O_3_ on SiO_2_ for CO methanation from syngas. Catal. Today.

[16] Lovell E.C., Fuller A., Scott J., Amal R. (2016). Enhancing Ni-SiO_2_ catalysts for the carbon dioxide reforming of methane: Reduction-oxidation-reduction pre-treatment. Appl. Catal. B Environ.

[17] Rezaei M., Alavi S.M., Sahebdelfar S., Yan Z.F. (2006). Syngas production by methane reforming with carbon dioxide on noble metal catalysts. J. Nat. Gas Chem..

[18] Li D., Nakagawa Y., Tomishige K. (2011). Methane reforming to synthesis gas over Ni catalysts modified with noble metals. Appl. Catal. A-Gen..

[19] Wang F., Xu L., Zhang J., Zhao Y., Li H., Li H.X., Wu K., Xu Q.G., Chen W. (2016). Tuning the metal-support interaction in catalysts for highly efficient methane dry reforming reaction. Appl. Catal. B Environ..

[20] Zhang L.M., Li L., Li J.L., Zhang Y.H., Hu J.C. (2014). Carbon dioxide reforming of methane over nickel catalyst supported on MgO (111) nanosheets. Top. Catal..

[21] Sudarsanam P., Hillary B., Deepa D.K., Amin M.H., Mallesham B., Reddy B.M., Bhargava S.K. (2015). Highly efficient cerium dioxide nanocube-based catalysts for low temperature diesel soot oxidation: The cooperative effect of cerium- and cobalt-oxides. Catal. Sci. Technol..

[22] Srivastava R., Srinivas D., Ratnasamy P. (2005). CO_2_ activation and synthesis of cyclic carbonates and alkyl/aryl carbamates over adenine-modified Ti-SBA-15 solid catalysts. J. Catal..

[23] Kalapathy U., Proctor A., Schultz J. (2000). A simple method for the production of pure silica from rice hull ash. Bioresour. Technol..

[24] Yu J., Ni Y., Zhai M. (2018). Simple solution-combustion synthesis of Ni-NiO@C nanocomposites with highly electrocatalytic activity for methanol oxidation. J. Phys. Chem. Solid..

[25] Kelekanjeri V.S.K.G., Carter W.B., Hampikian J.M. (2006). Deposition of α-alumina via combustion chemical vapor deposition. Thin Solid Films.

[26] Sun G.B., Hidajat K., Wu X.S., Kawi S. (2008). A crucial role of surface oxygen mobility on nanocrystalline Y_2_O_3_ support for oxidative steam reforming of ethanol to hydrogen over Ni/Y_2_O_3_ catalysts. Appl. Catal. B Environ..

[27] Zhu F., Zhang H., Yan X., Yan J., Ni M., Li X., Tu X. (2017). Plasma-catalytic reforming of CO_2_-rich biogas over Ni/γ-Al_2_O_3_ catalysts in a rotating gliding arc reactor. Fuel.

[28] Mei D., Ashford B., He Y.-L., Tu X. (2017). Plasma-catalytic reforming of biogas over supported Ni catalysts in a dielectric barrier discharge reactor: Effect of catalyst supports. Plasma Process. Polym..

[29] Akbari E., Alavi S.M., Rezaei M. (2017). Synthesis gas production over highly active and stable nanostructured Ni-MgO-Al_2_O_3_ catalysts in dry reforming of methane: effects of Ni contents. Fuel.

[30] Zhang R.-J., Xia G.-F., Li M.-F., Wu Y., Nie H., Li D.-D. (2015). Effect of support on the performance of Ni-based catalyst in methane dry reforming. J. Fuel Chem. Technol..

[31] Hao Z., Zhu Q., Jiang Z., Hou B., Li H. (2009). Characterization of aerogel Ni/Al_2_O_3_ catalysts and investigation on their stability for CH_4_-CO_2_ reforming in a fluidized bed. Fuel Process. Technol..

[32] Al-Fatesh A.S., Atia H., Ibrahim A.A., Fakeeha A.H., Singh S.K., Labhsetwar N.K., Shaikh H., Qasim S.O. (2019). CO_2_ reforming of CH_4_: Effect of Gd as promoter for Ni supported over MCM-41 as catalyst. Renew. Energy.

[33] Yang W., Liu H., Li Y., Wu H., He D. (2016). CO_2_ reforming of methane to syngas over highly-stable Ni/SBA-15 catalysts prepared by P123-assisted method. Int. J. Hydrogen Energy.

